# Medical Error Prevalence, Nursing Power, and Structural Empowerment: A Serial Mediation Analysis

**DOI:** 10.1155/2024/1554373

**Published:** 2024-04-25

**Authors:** Wafa'a Ta'an

**Affiliations:** Department of Community and Mental Health Nursing, Faculty of Nursing, Jordan University of Science and Technology, P.O. Box 3030, Irbid 22110, Jordan

## Abstract

**Aim:**

To investigate how structural empowerment and power may contribute to and predict the reduction of medical errors.

**Background:**

Medical errors threaten patient well-being, leading to adverse outcomes. Improving work conditions holds promise for reducing medical errors among nurses.

**Methods:**

A multisite correlational cross-sectional design was utilized. Data were completed by 375 nurses from four hospitals in Jordan. Data collection occurred between September and November 2023 using sociodemographic, structural empowerment, and medical error questionnaires. The study employed descriptive statistics, Pearson *r* correlation, and serial mediation analysis. Informed consent was obtained from each participant.

**Results:**

Pearson *r* correlation revealed significant negative correlations between medical error and structural empowerment, formal power, and informal power. The conceptual framework was significant and predicted 16% of the variance in medical errors. The mediation analysis confirmed that formal power and informal power mediate the relationship between structural empowerment and medical error. *Conclusions and Implications*. This study sheds light on the intricate connection of structural empowerment, formal and informal power, and their collective impact on reducing medical errors. Understanding and addressing these dynamics allows nurses and administrators to achieve a culture of patient safety. Reduction of medical errors is paramount to a safe healthcare environment that prioritizes patient outcomes. Strategies should be fostered to enhance structural empowerment, refine formal power structures, and leverage the positive aspects of informal networks.

## 1. Background

Nurses assume a vital role in upholding the standards of high-quality healthcare services, with nurses often being the primary contact personnel in the patient care process [1]. The pursuit of quality care is a multifaceted endeavor, encompassing various critical aspects, one of which is the prevention of medical errors. Medical errors are defined as the failure of a planned action to be completed as intended or using the wrong plan to achieve an aim [2]. These errors can occur at any stage of the healthcare process and can have severe consequences for patients, including injury or death [3].

According to a register-based study of around 3 million certified deaths in the United States of America, 0.16–1.13% of all deaths were attributed to medical errors [4]. In Jordan, medical error is a significant concern, as it can lead to adverse patient outcomes and harm the healthcare system [5]. In 2018, the Jordanian Ministry of Health reported that medical errors accounted for 60% of all patient complaints, highlighting the urgent need to address this issue [6]. These errors pose a substantial threat to patient well-being, potentially leading to adverse outcomes, elevated healthcare expenses [7], and reduced patient satisfaction [8]. Consequently, understanding and mitigating medical errors have become top priorities in the healthcare field in Jordan.

Within the context of healthcare quality enhancement, structural empowerment has emerged as a pivotal factor [9]. Structural empowerment refers to the organizational structures related to work conditions and processes that enable employees to perform their jobs effectively, make decisions, and take responsibility for their actions [10]. It includes access to information, resources, and support from colleagues and supervisors, opportunities for professional growth and development, and a sense of control over one's work environment [11, 12]. In addition, formal power (job characteristics) and informal power (peer networking and professional relationships) are considered essential factors contributing to access to structural empowerment [13, 14]. Formal power is also referred to as the Job Activity Scale (JAS), and informal power is referred to as the Organizational Relationships Scale (ORS) [13].

Improving work conditions (i.e., structural empowerment and nursing power) holds promise for reducing medical errors among nurses [15]. However, it is noteworthy that the precise mechanism by which structural empowerment and nursing formal and informal power contribute to the prevention of medical errors has yet to be thoroughly investigated within the healthcare context in Jordan.

This study derives from the recognition of the substantial challenges that nurses encounter in various healthcare settings, including those within Jordan, as evidenced by prior research [16–19]. Research has been undertaken to explore the connection between structural empowerment and different nursing outcomes, such as job satisfaction [20, 21], formal and informal power [14], employment retention [22], and patient outcomes [23, 24]. However, there is a lack of studies examining the link between structural empowerment as well as nursing power and medical errors among nurses. Therefore, this study seeks to address this gap in the literature and provide valuable insights into the relationship between these variables.

The theoretical framework underpinning this study is derived from Kanter's theory of structural empowerment [11]. Kanter theorized that structural empowerment could be achieved through four organizational structures: opportunity, information, support, and resources [11]. Work conditions demonstrated by structural empowerment have been proven to enhance nursing performance [25]. Nursing powers such as formal power (job characteristics) and informal power gained from peer networking and professional relationships were also found to positively impact nurses' performance and patient care quality [14]. The above-mentioned literature suggests that formal and informal power may mediate the relationship between structural empowerment and medical errors among nurses.

**Figure 1:**
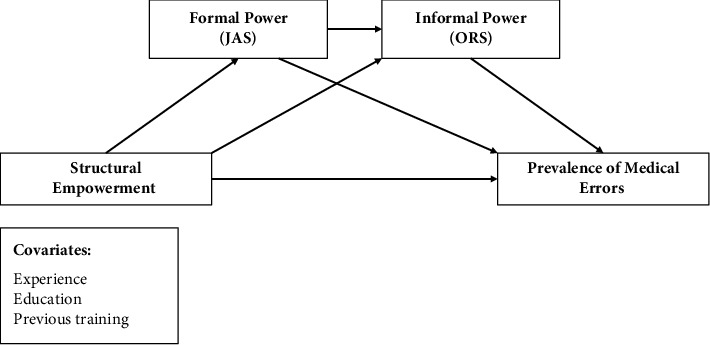
The study conceptual framework.

In this study, a conceptual framework was developed based on Kanter's theory of structural empowerment hypothesizing that structural empowerment, formal power, and informal power can impact nursing performance in terms of lowering the prevalence of medical errors (Figure 1). The study seeks to gain insights into how conducive structural empowerment and power may contribute to and predict the reduction of medical errors and eventually enhance patient safety within the healthcare context.

## 2. Methods

### 2.1. Design and Settings

A multisite descriptive correlational cross-sectional design was utilized to examine the impact of structural empowerment and support on the prevalence of medical errors among Jordanian nurses as proposed by the study's conceptual framework. The study occurred in four hospitals in Northern Jordan using convenience sampling techniques.

### 2.2. Sampling and Participants

The study's sample size was determined using Hulley et al.'s [26] equation. The calculation is total sample size = *N* = [(*Zα* + *Zβ*)/C]2 + 3), and alpha (two-tailed) was 0.05. The beta was 0.05, and the correlation coefficient was 0.20. According to Hulley et al. [26], sample size calculation using a correlation coefficient is common in clinical research such as nursing. A correlation coefficient represents the proportion of the spread (variance) in the measured variable that results from its linear association with the other variable(s). A small coefficient value of less than 0.3 can be used when a large sample size is recruited. The calculation resulted in 319 participants. A 30% increase in the calculated sample size (yielding 432) was made to ensure acquiring the minimum required sample size and to account for incomplete questionnaires. The inclusion criteria were nurses, full-time employment, and having a minimum of one year of experience to ensure their orientation to the organizational work environment and sufficient practical experience to reflect on. The final sample encompassed 375 nurses who had completed the questionnaire with a response rate of 86.8%.

### 2.3. Measures

Participants' data were collected using self-reported questionnaires including sociodemographic, medical error, structural empowerment, formal power, and informal power questionnaires.

#### 2.3.1. Sociodemographic Questionnaire

The sociodemographic questionnaire included questions on age, gender, educational level, years of experience, unit/department, and training opportunities.

#### 2.3.2. Medical Error Survey

The medical error survey was used to measure medical error prevalence [5]. This survey includes questions related to the frequency and extent of medical errors in health care. Participants were asked to report the occurrence of various types of errors, such as medication errors, patient falls, positioning or bed ulcers, blood transfusion errors, iatrogenic infections, Foley's catheter errors, communication failures, and misconnections of nasogastric (NG) and ventilator tubes. The Cronbach's coefficient of the medical error prevalence questionnaire was 0.92 in this study.

#### 2.3.3. Conditions for Work Effectiveness Questionnaire-II (CWEQ-II)

The Condition for Workplace Effectiveness Questionnaire-II (CWEQ-II) was also used to measure structural empowerment, formal support, and informal support [13]. The CWEQ-II contains four domains of structural empowerment (opportunity, resources, information, and support), formal power (Job Activity Scale: JAS), and informal power (Organization Relationships Scale: ORS). The study utilized the Arabic version and developed and validated by Ta'an et al. [27] with a Cronbach coefficient of 0.95 for the total questionnaire and 0.83–0.89 for the subscales.

### 2.4. Data Collection and Ethical Considerations

Ethical approval was obtained from the investigators' Institutional Research Board. Data collection occurred between September and November 2023. A trained research assistant provided potential participants with the study's purpose and information. Participants were assured that their anonymity and confidentiality would be preserved, and they could withdraw from the study at any time without penalty. After screening the potential participants for eligibility, nurses were provided with envelopes containing the study's questionnaires. Informed consent was obtained from each participant. Upon returning the questionnaires, participants were kindly asked to check the completeness of their responses to avoid unintentional missing data.

### 2.5. Data Analysis

The statistical package of social sciences (SPSS) for Windows, Version 27 (Armonk, NY), was used for the analysis. The study employed descriptive statistics and Pearson *r* correlation. In addition, serial mediation analysis using the Andrew Hays Process Macro-Model 6 was performed to test the conceptual model, suggesting that structural empowerment, formal power, and informal power will relate to the prevalence of medical errors among nurses in several direct and indirect pathways as illustrated in Figure 1. To account for other potential influencing factors, education, experience, and previous training were entered into the model as potential covariates. In the serial mediation analysis, 5,000 bootstrap resamples and a confidence interval of 95% were used to evaluate the significance of the results. The results are considered significant if “Zero” was not included in the upper to lower limits of the 95% confidence interval as recommended by Hayes [28]. Prior to conducting the main analysis of mediation, several assumptions should be made. The variables in the model need to be continuous, have linear relationships, not show multicollinearity, and be approximately normally distributed. The study data met the assumptions; the variables are continuous with linear relationships, and the normality was assessed using Shapiro–Wilk and Kolmogorov–Smirnov tests which resulted in nonsignificant results demonstrating normal distribution. There was no evidence of multicollinearity, as assessed by tolerance values greater than 0.1.

## 3. Results

### 3.1. Descriptive Results

A total of 375 nurses completed and returned the study questionnaires, with the majority being female (70%). Those who held a Bachelor's degree in nursing (BScN) represented 86% of the sample. As Table 1 shows, the mean age was 37 (SD = 6.2) years and experience averaged 12.7 (SD = 6.6) years. Only 46.4% of the nurses confirmed receiving training on medical error prevention, while 62.4% reported not receiving training. Participants were working in various units with the highest frequency in general units such as medical and surgical units (*n* = 234, 62.4%).

Table 2 presents the descriptive results of the participants' scores of structural empowerment (total and subscales), formal and informal power, and the prevalence of medical error based on actual responses. Scores of formal power, informal power, structural empowerment, and all of its subscales fell within moderate empowering work conditions based on the categorization proposed by the developer of CWEQ-II [13]. Participants reported a mean prevalence of medical errors of 22.43 (SD = 7.57).

The prevalence of medical errors was further categorized into low, moderate, and high levels. The results indicated that 332 nurses (88.5%) perceived medical errors as low, while 43 (11.5%) perceived them at a moderate level. This distribution indicates that while most nurses perceive only a low occurrence of medical errors in their work environment, there is still a notable proportion experiencing these errors at a moderate level.

### 3.2. Bivariate Correlations

Pearson *r* correlation analysis was performed to evaluate the relationships between the study's main variables among the participants. The analysis revealed that the prevalence of medical errors was significantly and negatively correlated with structural empowerment (*r* = −0.23, *p* < 0.05), opportunity (*r* = −0.104, *p* < 0.01), information (*r* = −0.244, *p* < 0.05), support (*r* = −0.195, *p* < 0.01), resources (*r* = −0.218, *p* < 0.01), formal power (*r* = −0.22, *p* < 0.01), and informal power (*r* = −0.375, *p* < 0.01). Noteworthy, these results indicate that as structural empowerment and nursing power increase, the prevalence of medical errors decreases.

### 3.3. Serial Mediation Analysis

To shed light on the specific mechanisms by which work condition factors regress into the outcome variable (the prevalence of medical error), the study's conceptual model was tested using Andrew Hays Process Macro-Model 6. The prevalence of medical errors was inserted as the outcome variable and structural empowerment was the dependent variable, whereas formal and informal powers were the mediators. Education, experience, and previous training were considered potential covariates. The model was significant (*F* (3,368) = 11.67, *p* < 0.001) and predicted 16% of the variance in medical errors.

The analysis under model 6 examined the effect of structural empowerment on medical errors through direct and indirect paths. The indirect paths include the effect of structural empowerment through (1) formal power, (2) informal power, and (3) formal and informal power. The analysis showed that the effect of structural empowerment on medical errors was not significant through the direct path. The effects of structural empowerment on medical errors through “informal power” and “formal and informal powers” were significant. However, the indirect path through “formal power” was not statistically significant (Table 3).

## 4. Discussion

The current study aimed to fill a gap in the literature concerning medical errors and their associated factors among Jordanian nurses. To achieve this goal, this study examined the complex relationships among study variables in predicting medical errors. More specifically, in this study, we examined whether informal and formal power would serially mediate the effect of structural empowerment on medical errors by controlling for experience, education, and previous training. Thus, the findings provide valuable insights into the ongoing discourse on patient safety and organizational dynamics within the healthcare sector in Jordan.

Nurses in this study perceived structural empowerment as moderate on average, with the highest scores in access to organizational resources and the lowest in access to information. Moderate structural empowerment was similarly reported in a cross-country study conducted in Spain and the United Kingdom [29] and in a Jordanian study [25]. However, developing empowering organizational structures is essential to foster nurses' performance, as Fragkos et al. [12] recommended. An empowered nurse can be best equipped to provide high-quality, error-free care.

In this study, more than half of the nurses did not receive training regarding medical error prevention, which is consistent with a previous study conducted in Jordan [5]. This result is alarming, taking into consideration that medical error is a major cause of death [4] and that about two-thirds of global medical error rates take place in low-middle-income countries [30]. Furthermore, a high percentage of nurses reported a low level of medical error prevalence. This finding provides crucial insights into the extent of medical errors in the nursing field in Jordan, highlighting the need for training opportunities on patient safety and error reduction strategies.

As this study showed, the prevalence of medical errors is disproportionally related to structural empowerment components and nursing power. This indicated that organizational effort could be invested in improving nurses' access to empowerment structures to mitigate medical errors. A supportive and empowering organizational structure can be achieved through fostering open communication, professional development opportunities, and shared decision-making [31]. Consequently, healthcare professionals working in such an environment are better equipped to provide safe and effective patient care [32]. The negative correlation observed between structural empowerment and the prevalence of medical errors is consistent with previous research, emphasizing the importance of nurturing organizational structures that promote employee engagement and autonomy [15]. As expected, nurses' experience was found to decrease the prevalence of medical errors. However, it is an unmodifiable factor, but a great utilization can be made to engage experienced nurses in developing medical error prevention strategies.

The results revealed that nursing “informal power” and “formal and informal powers” paths mediated the relationship between structural empowerment and medical errors. This is the first study to show that the reduction of medical error prevalence not only requires improved access to empowerment structures but also necessitates greater nursing power in formal and informal forms. This result resonates with a previous study that declared that the interplay between structural empowerment and nursing power does impact nurses' performance [20]. However, the previously mentioned study recommended future research that sheds light on this complex relationship and considers critical components of care service quality, including the reduction of medical errors.

Nursing formal power within healthcare institutions can be improved by enhancing job characteristics in terms of visibility, flexibility, creativity, and decision-making that align with organizational missions and goals. Nurses with power are more likely to sense their value in their organizations, allowing them to be more committed to improving care quality [33]. On the other hand, informal power emerges from the relationships and network of alliances among healthcare professionals [14]. For nurses, informal power plays a crucial role in shaping the overall institutional culture and functioning, specifically in the fields of communication and collaboration. This research revealed that informal power synergizes the effect of structural empowerment in decreasing medical errors. This was emphasized by a previous study, which showed that attitudes and practices concerning medical errors are affected by the medical hierarchy, and increasing nurses' power can encourage them to speak up and better collaborate to prevent harmful situations [34].

## 5. Limitations and Delimitations

To improve the transparency and generalizability of the study, it is important to address the limitations of this study. The study utilized a self-reported questionnaire to collect data. This may be threatened by social desirability bias, restricting participants' responses in critical areas such as medical errors. However, the participants were assured that their anonymity and confidentiality would be preserved to counteract this particular threat.

The study also utilized a convenience sampling approach. This may affect the generalizability of the study findings. Therefore, a multisite approach was used to investigate the study concepts and relationships of a wide range of nurses. By expecting and then proactively addressing these potential biases, the core of this study's findings yielded important recommendations and implications for nursing education, practice, research, and administration.

## 6. Conclusions and Implications

In conclusion, our study sheds light on the intricate connection of structural empowerment, formal and informal power, and their collective impact on reducing medical errors. By understanding and addressing these dynamics, nurses and administrators within healthcare organizations can move closer to achieving a culture of safety that prioritizes patient well-being and optimal healthcare outcomes.

This study confirms that while structural empowerment is important to the best care practice for reducing medical errors, nursing formal and informal power can facilitate effective communication and information sharing in medical error reduction. Our study recommends leveraging nursing autonomy and power as well as informal networks and professional alliances for positive outcomes. This can be achieved by ensuring that these networks enhance nurses' access to information, support, resources, and opportunity structures.

The implications of this study extend to healthcare practitioners, administrators, and policymakers. For example, strategies for enhancing structural empowerment, refining formal power structures, and leveraging the positive aspects of informal networks can be enacted. This contributes to a safer healthcare environment, as the current study illustrates. Future research should delve deeper into the specific mechanisms through which these factors interact and identify targeted interventions to reduce the prevalence of medical errors in the future.

Educational opportunities need to be enhanced through informal communication. A practical example is providing short educational materials on successful medical reduction strategies and/or success stories that are easily digested and shared through group media. On the other hand, an anonymous errors reporting forum may be established to help nurses raise awareness of the most prevalent medical error types to help nurses and their managers tackle these issues in a blame-free environment.

Modern training and educational opportunities can be utilized to reduce the prevalence of medical errors. Informal power should not be underestimated when creating such opportunities. For example, online anonymous forums may be helpful. However, future interventional research is warranted to uncover the effectiveness of such interventions. The hierarchical structure of healthcare institutions has been long criticized as healthcare institutions are dynamic and challenging. Therefore, effective empowerment can only be achieved through increasing nursing power to achieve the ultimate goal of medical error-free care services successfully.

## Figures and Tables

**Table 1:** Sociodemographic characteristics of participants (*N*  = 375).

Variable	Minimum	Maximum	Mean	SD
Age in years	22.00	55.00	36.96	6.18
Years of experience	1.00	28.00	12.73	6.56

Variable	Category		Frequency	Percentage

Gender	Male		114	30.4
	Female		261	69.6

Educational level	BScN		323	86.1
	Postgraduate studies		52	13.9

Department	Critical care		60	16.0
	General units		234	62.4
	Emergency departments		17	4.5
	Units (ex., dialysis, and cath lab)		63	16.8
	Outpatient clinics		1	0.3

Received training on medical error prevention	Yes		141	37.6
	No		234	62.4

**Table 2:** Descriptives of the main study variables (*N*  = 375).

Variable	Minimum	Maximum	Mean	SD
Opportunity subscale	1.00	5.00	3.07	0.99
Information subscale	1.00	5.00	2.90	1.01
Support subscale	1.00	5.00	3.12	0.95
Resources subscale	1.00	5.00	3.15	0.90
Structural empowerment	4.00	20.00	12.26	3.18
Formal power (JAS)	1.00	5.00	2.64	0.98
Informal power (ORS)	1.00	5.00	3.21	0.92
Medical error prevalence	11.00	47.00	22.43	7.57

**Table 3:** Serial mediation analysis summary.

The direct effect of structural empowerment on the prevalence of medical error						
Path	Effect	SE	*t*	*p*	LLCI	ULCI
S.E. ⟶ M.E. prevalence	0.089	0.177	0.503	0.616	−0.259	0.437

Indirect effects of structural empowerment on the prevalence of medical error						
Paths	Effect	SE	LLCI	ULCI		

S.E. ⟶ F.P. ⟶ M.E. prevalence	0.0486	0.1166	−0.1848	0.2753		
S.E. ⟶ I.P. ⟶ M.E. prevalence	−0.4621	0.0969	−0.6627	−0.2851		
S.E. ⟶ F.P. ⟶ I.P. ⟶ M.E. prevalence	−0.2176	0.0578	−0.3407	−0.1158		
Total	−0.631	0.149	−0.922	−0.343		

S.E. structural empowerment, F.P. formal power, I.P. informal power, M.E. medical error.

## Data Availability

The data that support the findings of this study are available from the author with permission from the Jordan University of Science and Technology.
